# Supplementary material for: Touch-sensitive stamens enhance pollen dispersal by scaring away visitors
Source: eLife. 2022 Oct 11;11:e81449. doi: 10.7554/eLife.81449 (PMC9555859; doi:10.7554/eLife.81449)
Supplement: Supplementary file 1. [file elife-81449-supp1.docx]

**Table S1.** Floral traits (mean ± SE) and duration of stamen movements in *Berberis jamesiana*, *B. julianae*, *B. forrestii*, and *Mahonia bealei*. Different superscript letters indicate significant differences; N = number of sampled flowers; － not measured.

| Floral traits | *Berberis julianae* | N | *Berberis jamesiana* | N | *Berberis forrestii* | N | *Mahonia bealei* | N | Wald χ^2^ | P |
| --- | --- | --- | --- | --- | --- | --- | --- | --- | --- | --- |
| Corolla diameter (mm) | 12.75^a^ ± 0.40 | 20 | 8.37^b^ ± 0.25 | 20 | 6.34^c^ ± 0.14 | 30 |  |  | 324.66 | **0.000** |
| Petal length (mm) | 6.10^a^ ± 0.07 | 20 | 4.78^b^ ± 0.07 | 20 | 2.47^c^ ± 0.07 | 30 |  |  | 1406.29 | **0.000** |
| Anther-stigma distance (mm) | 1.59^a^ ± 0.08 | 20 | 0.96^b^ ± 0.07 | 20 | 0.94^b^ ± 0.06 | 30 |  |  | 52.85 | **0.000** |
| Pistil height (mm) | 5.48^a^ ± 0.06 | 20 | 3.34^b^ ± 0.08 | 20 | 2.28^c^ ± 0.06 | 30 |  |  | 642.982 | **0.000** |
| Stamen length (mm) | 4.81^a^ ± 0.07 | 20 | 2.69^b^ ± 0.09 | 20 | 2.67^b^ ± 0.07 | 30 |  |  | 303.741 | **0.000** |
| Filament length (mm) | 3.33 ± 0.06 | 20 | － |  | － | － |  |  | － |  |
| Nectar volume per flower (μL) | 1.20 ± 0.03 | 20 | － |  | － | － |  |  | － |  |
| Pollen grains per flower | 7323^a^ ± 78 | 20 | 6810^b^ ± 156 | 20 | － | － |  |  | 8.478 | **0.004** |
| Ovule number per flower | 3.10^a^ ± 0.07 | 20 | 2.0^b^ ± 0.0 | 20 | 2.03^b^ ± 0.03 | 30 |  |  | 495.75 | **0.000** |
| Inward movement time (s) | 0.44^a^ ± 0.02 | 10 | 0.17^b^ ± 0.02 | 7 | 0.23^b^ ± 0.04 | 5 | 0.09^c^ ± 0.01 | 10 | 225.22 | **0.000** |
| Interval time (s) | 62.21^a^ ± 3.08 | 10 | 13.19^c^ ± 0.55 | 7 | 21.48^b^ ± 3.56 | 5 | 3.46^d^ ± 0.71 | 10 | 466.46 | **0.000** |
| Outward movement time (s) | 227.70^a^ ± 10.06 | 10 | 110.37^c^ ± 6.64 | 7 | 155.31^b^ ± 14.07 | 5 | 7.74^d^ ± 1.96 | 10 | 456.48 | **0.000** |
