# Supplementary material for: Touch-sensitive stamens enhance pollen dispersal by scaring away visitors
Source: eLife. 2022 Oct 11;11:e81449. doi: 10.7554/eLife.81449 (PMC9555859; doi:10.7554/eLife.81449)
Supplement: Supplementary file 2. — Behaviors of the bees and flies visiting Berberis julianae, focusing on visitation rates (visits per flower per hour, mean ± SE), visits per flower, handling time, number of stamens touched, nectar volume remaining per flower, pollen grains removed, pollen grains deposited, and pollen transfer efficiency. [file elife-81449-supp2.docx]

**Table S2.** Behaviours of the bees and flies visiting *Berberis julianae*, focusing on visitation rates (visits per flower per hour, mean ± SE), visits per flower, handling time, number of stamens touched, nectar volume remaining per flower, pollen grains removed, pollen grains deposited, and pollen transfer efficiency. Significant differences are shown with superscript letters.

| Family (Order) | Visitor species | Visitation rates in the field | | | Reward | Visits to flowers | Handling time per flower (s) | Stamens touched | Remaining nectar (μL) | Pollen | | |
| --- | --- | --- | --- | --- | --- | --- | --- | --- | --- | --- | --- | --- |
|  |  | 2019  (n = 20 h) | 2020 (n = 20 h) | 2021  (n = 35 h) |  |  |  |  |  | removal | receipt | transfer efficiency |
| Apidae (Hymenopt.) | *Apis cerana* | 0.200^ab^ ± 0.046 | 0.715^a^ ± 0.079 | 0.535^a^ ± 0.056 | Nectar | 0.9^a^ ± 0.2 | 5.614^c^ ± 0.606 | 2.5^a^ ± 0.2 | 0.92^b^ ± 0.02 | 1100^b^ ± 99 | 107^b^ ± 16 | 0.121^a^ ± 0.023 |
|  | Anthophorids | 0.449^a^ ± 0.168 | 0.012^b^ ± 0.009 | 0.023^b^ ± 0.015 | Nectar | 0.5^a^ ± 0.1 | 5.017^c^ ± 0.661 | 2.3^a^ ± 0.1 | 0.91^b^ ± 0.02 | 2202^a^ ± 184 | 226^a^ ± 21 | 0.129^a^ ± 0.021 |
| Syrphidae (Diptera) | *Meliscaeva* spec. | 0.024^c^ ± 0.006 | 0.004^b^ ± 0.002 | 0.014^b^ ± 0.005 | Pollen, nectar | 0.6^a^ ± 0.1 | 29.905^a^ ± 3.432 | 1.7^b^ ± 0.2 | 1.03^a^ ± 0.02 | 459^d^ ± 61 | 24^c^ ± 6 | 0.057^b^ ± 0.010 |
|  | *Rhingia campestris* | 0.019^cd^ ± 0.009 | 0.002^b^ ± 0.001 | 0.005^b^ ± 0.004 | Pollen, nectar | 0.7^a^ ± 0.2 | 22.232^b^ ± 3.823 | 1.9^b^ ± 0.1 | 1.02^a^ ± 0.02 | 742^c^ ± 90 | 40^c^ ± 8 | 0.063^b^ ± 0.015 |
| Syrphidae (Diptera) | *Eristalis* | 0.017^d^ ± 0.011 | 0.004^b^ ± 0.003 | 0.003^b^ ± 0.001 | Nectar |  |  |  |  |  |  |  |
| Calliphoridae (Diptera) | *Calliphora* | 0.007^d^ ± 0.004 | 0.004^b^ ± 0.003 | 0.001^b^ ± 0.001 | Pollen, nectar |  |  |  |  |  |  |  |
| Apidae (Hymenopt.) | *Bombus trifasciatus* | 0.002^d^ ± 0.001 | 0.001^b^ ± 0.001 | － | Nectar |  |  |  |  |  |  |  |
|  | Wald χ^2^ | 40.673 | 85.356 | 141.506 |  | 4.563 | 175.358 | 19.152 | 37.863 | 127.701 | 119.597 | 38.308 |
|  | df | 6 | 6 | 5 |  | 3 | 3 | 3 | 3 | 3 | 3 | 3 |
|  | *P* | <**0.001** | <**0.001** | <**0.001** |  | 0.207 | <**0.001** | <**0.001** | <**0.001** | <**0.001** | <**0.001** | <**0.001** |
