# Supplementary material for: Touch-sensitive stamens enhance pollen dispersal by scaring away visitors
Source: eLife. 2022 Oct 11;11:e81449. doi: 10.7554/eLife.81449 (PMC9555859; doi:10.7554/eLife.81449)
Supplement: Supplementary file 3. [file elife-81449-supp3.docx]

**Table S3.** G–test of independence confirming that *Berberis julianae* flowers with mobile stamens (SM flowers) donated pollen to more recipient flowers at three distance classes than did flowers with experimentally immobilized stamens (SI flowers). Numbers of sampled flowers are pooled from four runs of the experiment.

| Pollen donors | Numbers of pollen-recipients/number of total flowers | | | |
| --- | --- | --- | --- | --- |
|  | <50 cm | 50 to 100 cm | >100 cm | All |
| SM flowers | 25/338 | 19/434 | 6/222 | 50/994 |
| SI flowers | 10/395 | 4/338 | 0 | 14/733 |
| G | 9.645 | 7.439 |  | 12.421 |
| *P* | **0.001899** | **0.006382** |  | **0.000425** |
