# Supplementary material for: Touch-sensitive stamens enhance pollen dispersal by scaring away visitors
Source: eLife. 2022 Oct 11;11:e81449. doi: 10.7554/eLife.81449 (PMC9555859; doi:10.7554/eLife.81449)
Supplement: Supplementary file 4. [file elife-81449-supp4.docx]

**Table S4.** G–test of independence confirming that *Berberis jamesiana* flowers with mobile stamens (SM flowers) donated pollen to more recipient flowers at two distance classes than did flowers with experimentally immobilized stamens (SI flowers). Numbers of sampled flowers are pooled from four runs of the experiment.

| Pollen donors | Number of pollen recipients/total number of flowers | |
| --- | --- | --- |
|  | <25 cm | >25 cm |
| SM flowers | 13/60 | 7/60 |
| SI flowers | 9/46 | 1/56 |
| G | 0.07 | 4.961 |
| *P* | 0.791 | **0.026** |
