# Supplementary material for: Touch-sensitive stamens enhance pollen dispersal by scaring away visitors
Source: eLife. 2022 Oct 11;11:e81449. doi: 10.7554/eLife.81449 (PMC9555859; doi:10.7554/eLife.81449)
Supplement: Supplementary file 5. [file elife-81449-supp5.docx]

**Table S5.** Concentrations of berberine (mean ± SE) in different tissues of *Berberis julianae* under Generalized Linear Models. Different superscript letters indicate significant differences in that tissue (Wald χ^2^ = 46885.3, P < 0.001, df = 3).

| Tissues | Concentrations of berberine (mg/g) | N |
| --- | --- | --- |
| Leaf | 0.0519^a^ ± 0.0001 | 10 |
| Petal | 0.0357^b^ ± 0.0001 | 10 |
| Pollen | 0.0202^c^ ± 0.0005 | 7 |
| Nectar | 0.0 ± 0.0 | 10 |
